# Bepridil is potent against SARS-CoV-2 in vitro

**DOI:** 10.1073/pnas.2012201118

**Published:** 2021-02-17

**Authors:** Erol C. Vatansever, Kai S. Yang, Aleksandra K. Drelich, Kaci C. Kratch, Chia-Chuan Cho, Kempaiah Rayavara Kempaiah, Jason C. Hsu, Drake M. Mellott, Shiqing Xu, Chien-Te K. Tseng, Wenshe Ray Liu

**Affiliations:** ^a^The Texas A&M Drug Discovery Laboratory, Department of Chemistry, Texas A&M University, College Station, TX 77843;; ^b^Department of Microbiology and Immunology, University of Texas Medical Branch, Galveston, TX 77555;; ^c^Department of Biochemistry and Biophysics, Texas A&M University, College Station, TX 77843;; ^d^Center of Biodefense and Emerging Disease, University of Texas Medical Branch, Galveston, TX 77555;; ^e^Institute of Biosciences and Technology, College of Medicine, Texas A&M University, Houston, TX 77030;; ^f^Department of Translational Medical Sciences, College of Medicine, Texas A&M University, Houston, TX 77030;; ^g^Department of Molecular and Cellular Medicine, College of Medicine, Texas A&M University, College Station, TX 77843

**Keywords:** COVID-19, SARS-CoV-2, main protease, bepridil, drug repurposing

## Abstract

Guided by a computational docking analysis, about 30 Food and Drug Administration/European Medicines Agency (FDA/EMA)-approved small-molecule medicines were characterized on their inhibition of the severe acute respiratory syndrome coronavirus 2 (SARS-CoV-2) main protease (M^p^^ro^). Of these small molecules tested, six displayed a concentration that inhibits response by 50% (IC_50_) value below 100 μM in inhibiting M^p^^ro^, and, importantly, three, that is, pimozide, ebastine, and bepridil, are basic molecules that potentiate dual functions by both raising endosomal pH to interfere with SARS-CoV-2 entry into the human cell host and inhibiting M^p^^ro^ in infected cells. A live virus-based modified microneutralization assay revealed that bepridil possesses significant anti−SARS-CoV-2 activity in both Vero E6 and A459/ACE2 cells in a dose-dependent manner with low micromolar effective concentration, 50% (EC_50_) values. Therefore, the current study urges serious considerations of using bepridil in COVID-19 clinical tests.

## Significance

Guided by a computational docking analysis, we experimentally characterized about 30 FDA/EMA-approved drugs on their inhibition of essential main protease of SARS-CoV-2, the pathogen of COVID-19. From this study, we discovered that bepridil, an antianginal medication, is potent against SARS-CoV-2. The antiviral analysis of bepridil indicated that it had low micromolar EC_50_ values in inhibiting SARS-CoV-2 in two highly permissive mammalian cell lines. Due to the urgent matter of COVID-19, our current study encourages further preclinical investigations of bepridil in animal models to clear its path for clinical uses in COVID-19 patients.

The current worldwide impact of the COVID-19 pandemic has been so profound that it is often compared to that of the 1918 influenza pandemic (1, 2). As of February 4, 2021, the total global COVID-19 cases had surpassed 100 million, among which more than 2 million had succumbed to death (3). Important political figures who have been diagnosed COVID-19 positive include the US president Donald Trump and the UK prime minister Boris Johnson. A modeling study has predicted that this pandemic will continue to affect everyday life, and the circumstances may require societies to follow social distancing until 2022 (4). Finding timely treatment options is of tremendous importance to alleviate catastrophic damages of COVID-19. However, the short time window that is required to contain the disease is extremely challenging for a conventional drug discovery process that requires typically many years to finalize a drug and therefore might not achieve its goal before the pandemic ceases. In January 2020, we did a comparative biochemical analysis between severe acute respiratory syndrome coronavirus 2 (SARS-CoV-2), the virus that has caused COVID-19, and SARS-CoV-1 that led to an epidemic in China in 2003, and proposed that remdesivir was a viable choice for the treatment of COVID-19 (5). We were excited to see that remdesivir was finally approved for emergency use in the United States and for use in Japan for people with severe symptoms. With only one medicine in stock that provides very limited benefits to COVID-19 patients (6), the virus may easily evade it, leaving us once again with no medicine to use. Given the rapid spread and the high fatality rate of COVID-19, finding alternative medicines is imperative. Drug repurposing stands out as an attractive option in the current situation. If an approved drug can be identified to treat COVID-19, it can quickly proceed to clinical trials and be manufactured at a large scale using its existing good manufacturing practice (GMP) lines. Previously, encouraging results were obtained from repurposing small-molecule medicines, including teicoplanin, ivermectin, itraconazole, and nitazoxanide (7–10). These antimicrobial agents showed antiviral activity against Ebola, Chikungunya, enterovirus, and influenza viruses, respectively (11). However, a common drawback of a repurposed drug is its low efficacy level. One way to circumvent this problem is to combine multiple existing medicines to accrue a synergistic effect. To be able to discover such combinations, breaking down the druggable targets of SARS-CoV-2 to identify drugs that do not cross-act on each other’s targets is a promising strategy. For example, a recent study showed that triple combination of interferon β-1b, lopinavir−ritonavir, and ribavirin was safe and superior to lopinavir−ritonavir alone for treating COVID-19 patients (12).

In our January paper (5), we recommended four SARS-CoV-2 essential proteins, including Spike, RNA-dependent RNA polymerase, the main protease (M^p^^ro^), and papain-like protease, as drug targets for the development of anti−COVID-19 therapeutics. Among these four proteins, M^p^^ro^ that was previously called 3C-like protease provides the most facile opportunity for drug repurposing, owing to the ease of its biochemical assays. M^p^^ro^ is a cysteine protease that processes itself and then cleaves a number of nonstructural viral proteins from two very large polypeptide translates that are made from the viral genomic RNA in the human cell host (13). Its relatively large active site pocket and a highly nucleophilic, catalytic cysteine residue make it likely to be inhibited by a host of existing and investigational drugs. Previous work has disclosed some existing drugs that inhibit M^p^^ro^ (14). However, complete characterization of existing drugs on the inhibition of M^p^^ro^ is not yet available. Since the release of the first M^p^^ro^ crystal structure, many computational studies have been carried out to screen existing drugs in their inhibition of M^p^^ro^, and many potent leads have been proposed (15–18). However, most of these lead drugs are yet to be confirmed experimentally. To investigate whether some existing drugs can potently inhibit M^p^^ro^, we have docked a group of selected Food and Drug Administration/European Medicines Agency (FDA/EMA)-approved small-molecule medicines to the active site of M^p^^ro^ and selected about 30 hit drugs to characterize their inhibition on M^p^^ro^ experimentally. Our results revealed that a number of FDA/EMA-approved small-molecule medicines have high potency in inhibiting M^p^^ro^, and bepridil inhibits cytopathogenic effect (CPE) induced by the SARS-CoV-2 virus in Vero E6 and A549/ACE2 cells with low micromolar effective concentration, 50% (EC_50_) values. Therefore, the current study encourages further preclinical testing of bepridil in animal models, paving the way to its clinical use against COVID-19.

## Results and Discussion

Jin et al. (14) released the first crystal structure of M^p^^ro^ on February 5, 2020. We chose this structure (Protein Data Bank ID code 6lu7) as the basis for our initial docking study. M^p^^ro^ has a very large active site that consists of several smaller pockets for the recognition of amino acid residues in its protein substrates. Three pockets that bind the P1, P2, and P4 residues in a protein substrate potentially interact with aromatic and large hydrophobic moieties (19). Although the P1′ residue in a protein substrate is a small residue such as glycine or serine, previous studies based on the same functional enzyme from SARS-CoV-1 showed that an aromatic moiety can occupy the site that originally bind the P1′ and P2′ residues in a substrate (20). Based on this analysis of the M^p^^ro^ structure, we selected 55 FDA/EMA-approved small-molecule medicines that have several aromatic or large hydrophobic moieties interconnected and did a docking analysis of their binding to M^p^^ro^. Some of the small-molecule medicines used in our docking study were previously reported in other computational studies (15–18). Autodock was the program we adopted for the docking analysis (21). The covalent ligand and nonbonded small molecules in the structure of 6lu7 were removed to prepare the protein structure for docking. Four residues, His41, Met49, Asnl42, and Glnl89, that have shown conformational variations in the SARS-CoV-1 enzyme were set flexible during the docking process. We carried out a genetic algorithm method with 100 runs to dock each small-molecule medicine to the enzyme. We collected the lowest binding energy from the total 100 runs for each small-molecule medicine and summarized them in Table 1. Among all 55 small-molecule drugs that we used in the docking study, 29 showed a binding energy lower than −8.3 kcal/mol. We chose these molecules (their structures are given in *SI Appendix*, Fig. S1) to do further experimental characterizations.

**Table 1.** Docking results of small-molecule medicines

Name	ΔG_binding_ (kcal/mol)	Name	ΔG_binding_ (kcal/mol)
Rimonabant*	−11.23	Bepridil*	−8.31
Tipranavir*	−10.74	Isoconazole	−8.15
Ebastine*	−10.62	Econazole	−8.14
Saquinavir*	−10.37	Eluxadoline	−8.12
Zopiclone*	−10.10	(R)-Butoconazole	−8.11
Pimozide*	−10.01	(S)-Butoconazole	−8.10
Pirenzepine*	−9.94	Atazanavir	−8.08
Nelfinavir*	−9.67	Cetirizine	−8.01
Doxapram*	−9.55	Efinaconazole	−8.01
Oxiconazole*	−9.18	Amprenavir	−7.99
Indinavir*	−9.13	Hydroxyzine	−7.99
Sertindole*	−9.04	(R)-Tioconazole	−7.98
Metixene*	−9.01	(R)-Carbinoxamine	−7.96
Fexofenadine*	−8.95	Armodafinil	−7.90
Lopinavir*	−8.91	Desipramine	−7.84
Sertaconazole*	−8.87	Ritonavir	−7.74
Reboxetine*	−8.86	Atomoxetine	−7.73
Ketoconazole*	−8.85	Sulconazole	−7.69
Duloxetine*	−8.79	Clotrimazole	−7.67
Isavuconazole*	−8.77	Dipyridamole	−7.67
Lemborexant*	−8.75	Phentolamine	−7.61
Oxyphencyclimine*	−8.74	(S)-Tioconazole	−7.48
Darunavir*	−8.72	Doxylamine	−7.33
Trihexphenidyl*	−8.72	(S)-Carbinoxamine	−7.21
Pimavanserin*	−8.69	Antazoline	−6.86
Clotiapine*	−8.57	Voriconazole	−6.76
Itraconazole*	−8.44	Fluconazole	−6.41
Clemastine*	−8.36		

* Compounds whose IC_50_ values were tested.

**Fig. 1.**
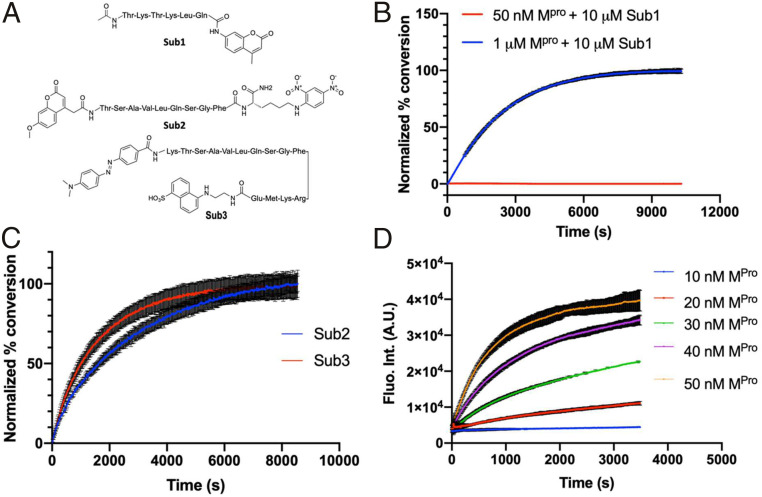
Activity of M^p^^ro^. (*A*) The structures of three substrates. (*B*) Activity of 50 nM M^p^^ro^ on 10 μM Sub1. (*C*) Activity of 50 nM M^p^^ro^ on 10 μM Sub2 and Sub3. The florescence signals are normalized for easy comparison. (*D*) Activity of different concentrations of M^p^^ro^ on 10 μM Sub3 plotted as fluorescence intenisty (fluo. int.) in arbitrary units (a.u.) vs time graph. All experiments were carried with three repeats, and data are presented as the average of three repeats in color, with error bars shown in black.

To express M^p^^ro^ for experimental characterizations of 29 selected small-molecule medicines, we fused the M^p^^ro^ gene to a superfolder green fluorescent protein (sfGFP) gene and a 6xHis tag at its 5′ and 3′ ends, respectively, in a pBAD-sfGFP plasmid (22) that we used previously in the laboratory. SfGFP is known to stabilize proteins when it is genetically fused to them (23). We designed a tobacco etch virus (TEV) protease cleavage site between sfGFP and M^p^^ro^ for the TEV-catalyzed proteolytic release of M^p^^ro^ from sfGFP after we expressed and purified the fusion protein. We placed the 6xHis tag right after the M^p^^ro^ C terminus. The addition of this tag was for straightforward purification with Ni-NTA resins. We expected that the TEV protease cleavage of sfGFP would activate M^p^^ro^ to cleave the C-terminal 6xHis tag so that a finally intact M^p^^ro^ protein would be obtained. We carried out the expression in *Escherichia coli* TOP10 cells. To our surprise, after expression, there was a minimal amount of the fusion protein that we were able to purify. The analysis of the cell lysate showed clearly the cleavage of a substantial amount of M^p^^ro^ from sfGFP. Since we were not able to enrich the cleaved M^p^^ro^ using Ni-NTA resins, the C-terminal 6xHis tag was apparently cleaved as well. TEV protease is a cysteine protease that cleaves after the Gln residue in the sequence Glu−Asn−Leu−Tyr−Phe−Gln−(Gly/Ser) (24). M^p^^ro^ is known to cleave the sequence Thr−Val−Leu−Gln−(Gly/Ser) (25). The two cleavage sites share the same P1 residue. It was evident in our expression work that M^p^^ro^ is able to efficiently cleave the TEV protease cutting site to maturate inside *E. coli* cells. According to a peptide library screening study, it is likely that M^p^^ro^ has a substrate promiscuity higher than what we have learned from the SARS-CoV-1 enzyme (25). In this study, activities of SARS-CoV-1 M^pro^ and SARS-CoV-2 M^pro^ enzymes were tested against a combinatorial substrate library. The results showed that both enzymes share a significant similarity in substrate specificity, but SARS-CoV-2 M^pro^ tolerates unnatural hydrophobic residues at the P2 position more than SARS-CoV-1 M^pro^. To purify the cleaved and maturated M^p^^ro^, we used ammonium sulfate to precipitate it from the cell lysate and then used the ion exchange and size exclusion chromatography to isolate it to more than 95% purity. We designed and synthesized a fluorogenic coumarin-based hexapeptide substrate (Sub1) and a fluorescence resonance energy transfer (FRET)-based decapeptide substrate (Sub2) and acquired a commercial FRET-based tetradecapeptide substrate (Sub3) (Fig. 1*A*). The test of enzyme activities on the three substrates indicated that the enzyme had low activity toward Sub1 (Fig. 1*B*) and its activity on Sub3 was higher than that on Sub2 (Fig. 1*C*) under our assay conditions. We subsequently used Sub3 in all the following inhibition analysis. To identify an optimal enzyme concentration for use in our inhibition analysis, we tested activities of different concentrations of M^p^^ro^ on 10 µM Sub3; the detected catalytic rate of the Sub3 cleavage was not proportional to the enzyme concentration (Fig. 1*D*). When the enzyme concentration decreased from 50 nM to 10 nM, the Sub3 cleavage rate dropped roughly proportionally to the square of the concentration decrease, characteristic of second-order kinetics. This observation supports previous claims that the enzyme needs to dimerize in order to be active (14). In all the following assays, 50 nM M^p^^ro^ and 10 µM Sub3 were used throughout.

**Fig. 2.**
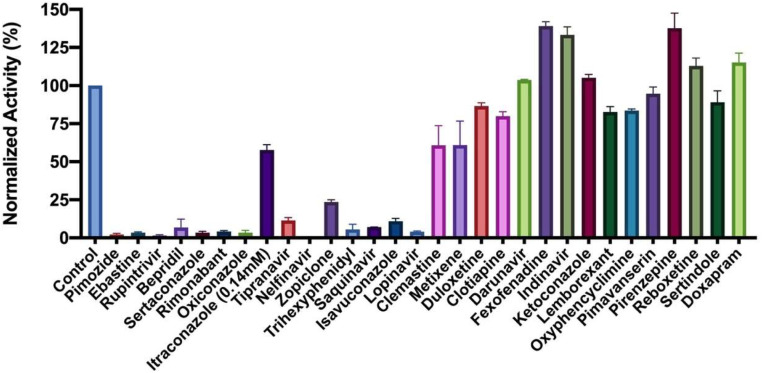
Initial screening of M^pro^ inhibition by 29 FDA/EMA-approved medicines and rupintrivir; 1 mM (0.14 mM for Itraconazole due to its low solubility in DMSO) was used for each inhibitor to perform the inhibition assay. Fluorescence intensity was normalized with respect to the control that had no small molecule provided. Triplicate experiments were performed for each compound, and the value was presented as mean ± SE.

We purchased all 29 small-molecule medicines from commercial providers without further purification and characterization. Rupintrivir is a previously developed 3C protease inhibitor (26). It has a key lactone side chain in the P1 residue that has a demonstrated role in tight binding to 3C and 3C-like proteases. Since it has been an investigational antiviral, we purchased it, as well, with a hope that it could be a potent inhibitor of M^p^^ro^. We dissolved most purchased small-molecule medicines in dimethyl sulfoxide (DMSO) to make 5 mM stock solutions and proceeded to use these stock solutions to test inhibition on M^p^^ro^. Except itraconazole that has low solubility in DMSO, all tested small-molecule medicines were diluted to a 1-mM final concentration in the inhibition assay conditions. We maintained 20% DMSO in the final assay condition to prevent small-molecule medicines from precipitating. The activity of M^p^^ro^ in 20% DMSO was 29% lower than that in a regular buffer (*SI Appendix*, Fig. S2) but satisfied our assay requirement, which is to have a fluorescence signal strong enough to produce a straight line for reliable and reproducible calculation of initial slopes. An M^p^^ro^ activity assay in the absence of a small-molecule medicine was set up as a comparison. Triplicate repeats were carried out for all tested small molecules and the control. The results presented in Fig. 2 displayed two easily discernable characteristics. First, about half of the tested compounds showed strong inhibition of M^p^^ro^ at the 1-mM concentration level (itraconazole was at 0.14 mM due to its low solubility in DMSO), supporting the practical use of a docking method in guiding the drug repurposing research of COVID-19. Second, several small-molecule medicines, including fexofenadine, indinavir, pirenzepine, reboxetine, and doxapram, clearly activated M^p^^ro^ (>15%). This was contrary to what the docking program predicted. This observation strongly suggests that frontline clinicians need to exhibit caution in repurposing medicines for COVID-19 patients before they are thoroughly investigated on influencing the SARS-CoV-2 biology. A not-well-understood drug might cause the already devastating symptoms in COVID-19 patients to progress. Although it is not the focus of the current study, the observation that M^p^^ro^ can be activated by existing drugs needs to be further investigated.

**Fig. 3.**
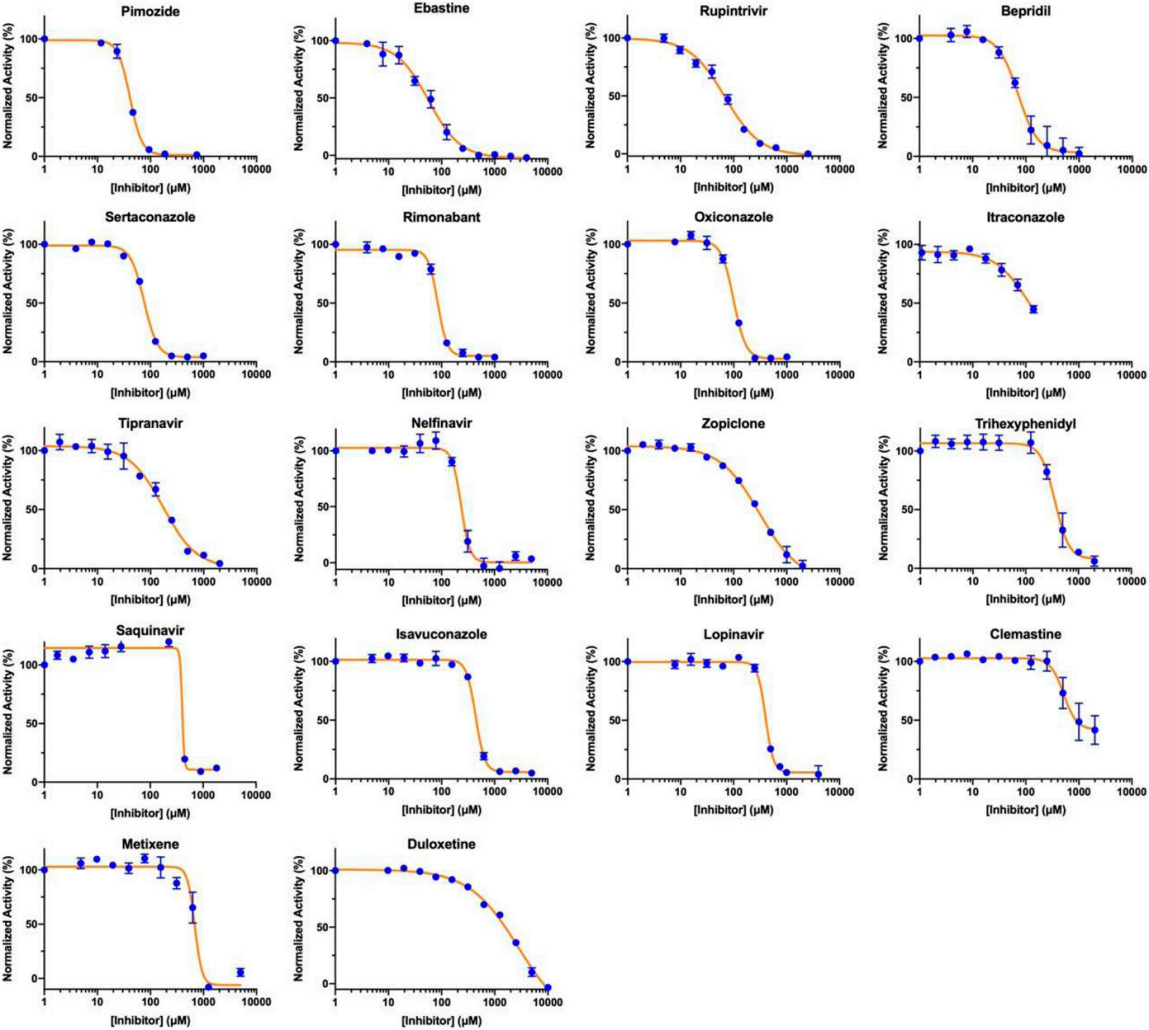
IC_50_ assays for 18 small-molecule medicines on their inhibition of M^pro^. Triplicate experiments were performed for each compound, and the IC_50_ value was presented as mean ± SE. GraphPad Prism 8.0 was used to perform data analysis.

We selected 17 small-molecule medicines and rupintrivir that displayed strong inhibition of M^p^^ro^, to conduct further characterizations of their concentration that inhibits response by 50% (IC_50_) values in inhibiting M^p^^ro^ by varying the small-molecule concentration from 1 µM to 10 mM. Results collectively presented in Fig. 3 identify that, of the 18 tested compounds, 7 had an IC_50_ value below 100 µM. These include pimozide, ebastine, rupintrivir, bepridil, sertaconazole, rimonabant, and oxiconazole. There is no strong correlation between calculated binding energies and determined IC_50_ values. This discrepancy can be explained by limited factors that were involved in the calculation. Pimozide, ebastine, and bepridil were the three most potent FDA/EMA-approved medicines, with IC_50_ values of 42 ± 2, 57 ± 12, and 72 ± 12 µM, respectively. Although rupintrivir is a covalent inhibitor that was developed specifically for 3C and 3C-like proteases, its IC_50_ value (68 ± 7 µM) is higher than that of pimozide and ebastine. The relatively low activity of rupintrivir in inhibiting M^p^^ro^ might be due to the change of the amide bond between the P2 and P3 residues to a methyleneketone. This conversion served to increase the serum stability of rupintrivir, but has likely eliminated a key hydrogen bonding interaction with M^p^^ro^ (14). The repurposing of HIV medicines for the treatment of COVID-19, particularly those targeting HIV1 protease, has been an area of much attention. In fact, the mixture of lopinavir and ritonavir was previously tested in China for the treatment of COVID-19 (27). The IC_50_ value of lopinavir in inhibiting M^p^^ro^ is about 500 µM, which possibly explains why this anti-HIV viral mixture demonstrated no significant benefit for treating patients. Nelfinavir was previously shown to have high potency in inhibiting the entry of SARS-CoV-2 into mammalian cell hosts. A cell-based study in inhibiting the SARS-CoV-2 entry indicated a 1-µM EC_50_ value (28, 29). However, our IC_50_ determination against M^p^^ro^ resulted in a value of 234 ± 5 µM, highlighting that nelfinavir likely inhibits another key SARS-CoV-2 enzyme or protein or interferes with key cellular processes that are required for the SARS-CoV-2 entry into host cells. These possibilities need to be studied further.

**Fig. 4.**
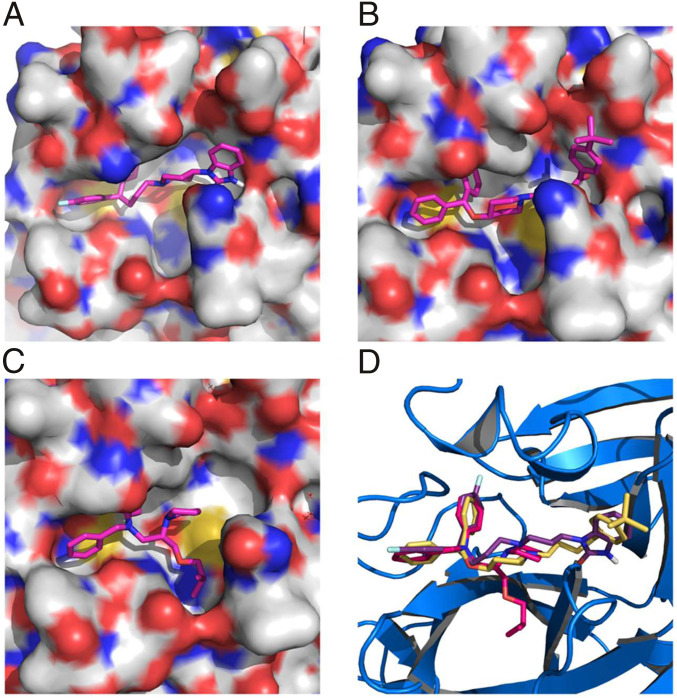
(*A*) Pimozide, (*B*) ebastine, (*C*) bepridil, and (*D*) their overlay in the active site of M^p^^ro^. The protein surface topography in *A−C* is presented to show the concaved active site.

Structurally, the two most potent medicines, pimozide and ebastine, share the same diphenylmethyl moiety. A spatially similar structure moiety *N*-phenyl-*N*-benzylamine exists in bepridil. Our docking results suggested the same binding mode for this similar structure moiety in all three drugs (Fig. 4). The two aromatic rings occupy the enzyme pockets that associate with the P2 and P4 residues in a substrate. This observation is in line with a crystallographic study that showed two aromatic rings with a single methylene linker bound to the active site of the SARS-CoV-1 enzyme (30). We believe that the inclusion of the diphenylmethyl moiety in structure−activity relationship studies of M^p^^ro^-targeting ligands will likely contribute to the identification of both potent and high cell-permeable M^p^^ro^ inhibitors. Fig. 3 also revealed large variations in Hill coefficients of IC_50_ curves for different small-molecule medicines (IC_50_ values and Hill coefficients are summarized in Table 2). Duloxetine and zopiclone gave the two highest Hill coefficients, with a gradual M^p^^ro^ activity decrease over an increasing inhibitor concentration. Conversely, saquinavir and lopinavir yielded the lowest Hill coefficients, with highly steep IC_50_ curves. There are three possible explanations for the large discrepancy in Hill coefficients. It could be attributed to different solubility of tested compounds. It is possible that a high DMSO percentage and a relatively high inhibitor concentration created phase transition for some inhibitors (31). A high Hill coefficient may also be due to different ligand-to-enzyme ratios when tested compounds bind to M^p^^ro^. An additionally possible reason is the coexistence of the M^p^^ro^ dimer and monomer in the assay conditions. A previous report showed a K_d_ value of the M^p^^ro^ dimerization as 2.5 µM (19). In theory, the catalytically active dimer species was present at a very low concentration in our assay conditions, leaving the catalytically inactive monomer species as the major form of M^p^^ro^. In this situation, the inhibitors that preferentially bind to the M^pro^ dimer and the inhibitors that have a higher affinity to the M^p^^ro^ monomer might yield different Hill coefficients. Although there is no report on the physiological concentration of M^p^^ro^ in infected cells, it is unlikely that it can reach 1 μM (34 ppm). Even at 1 μM, the majority of M^p^^ro^ is in its inactive monomeric form. Therefore, we believe that our assay conditions mimic physiological states of M^p^^ro^.

**Table 2.** IC_50_ and Hill coefficient values of 18 identified inhibitors

Name	IC_50_ (µM)	Hill slope
Pimozide	42 ± 2	3.1 ± 0.4
Ebastine	57 ± 12	1.5 ± 0.2
Rupintrivir	68 ± 7	1.4 ± 0.2
Bepridil	72 ± 3	2.9 ± 1.0
Sertaconazole	76 ± 2	3.5 ± 0.2
Rimonabant	85 ± 3	5.0 ± 0.4
Oxiconazole	99 ± 6	3.8 ± 0.4
Itraconazole	111 ± 35	1.6 ± 0.2
Tipranavir	180 ± 20	1.4 ± 0.2
Nelfinavir	234 ± 15	5.4 ± 1.0
Zopiclone	349 ± 77	1.2 ± 0.2
Trihexyphenidyl	370 ± 53	8.9 ± 6.4
Saquinavir	411 ± 6	26.8 ± 2.6
Isavuconazole	438 ± 11	5.2 ± 0.7
Lopinavir	486 ± 2	29.9 ± 2.4
Clemastine	497 ± 148	11.2 ± 7.3
Metixene	635 ± 43	8.7 ± 5
Duloxetine	3,047 ± 634	0.93 ± 0.07

**Fig. 5.**
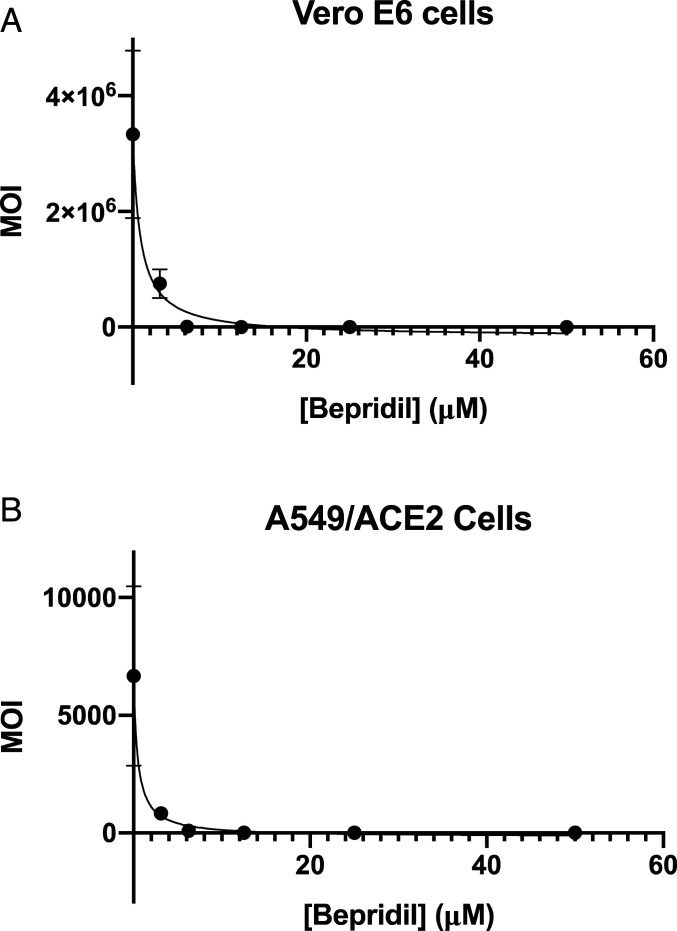
The SARS-CoV-2 inhibition by bepridil in (*A*) Vero E6 and (*B*) A549/ACE2 cells. Cells were incubated with different concentrations of bepridil and then infected with 0.5 MOI of SARS-CoV-2. Cells were let grow for 3 d for Vero E6 and 4 d for A549/ACE2 cells before their virus loads were determined. All experiments were performed in triplicate. Average MOIs with their SDs are presented.

The endocytic pathway has been proposed as a key step for the SARS-CoV-2 entry into host cells (32), making strategies that disrupt this process attractive therapeutic candidates for COVID-19. Based on this concept, hydroxychloroquine that has an ability to raise endosomal pH (33, 34) has been clinically investigated for treating COVID-19, albeit with close to no effects (35–37). From the chemistry point of view, our three lead compounds, pimozide, ebastine, and bepridil, share a similarity. They are all basic small molecules that can potentially raise endosomal pH (38). Among the three drugs, bepridil can be very interesting because it previously provided 100% protection from Ebola virus infections in mice at a dose of 12 mg/kg (39). Bepridil is a calcium channel blocker with significant antianginal activity. For patients with chronic stable angina, the recommended daily dose of bepridil is 200 mg to 400 mg (30). Mice administered with a bepridil dose as high as 300 mg⋅kg^−1^⋅d^−1^ did not show alteration in mating behavior and reproductive performance, indicating that bepridil has very low toxicity (40). Moreover, a previous study showed that bepridil can increase the pH of acidic endosomes (41). Administration of a high dose of bepridil may have dual functions to slow down the virus replication in host cells by both inhibiting M^p^^ro^ and raising the pH of endosomes. To demonstrate this prospect, we conducted a live virus-based microneutralization (MN) assay to evaluate efficacy of pimozide, ebastine, and bepridil in their inhibition of SARS-CoV-2 infection in a kidney epithelial cell line isolated from African green monkey (Vero E6) and human A549 cells stably transduced with human ACE2 viral receptor (A549/ACE2). We tested three medicines in a concentration range from 0.78 μM to 200 μM. CPE was clearly observable for pimozide and ebastine at all tested concentrations. However, bepridil prevented completely the SARS-CoV-2−induced CPE in Vero E6 and A549/ACE2 cells when its concentration reached 6.25 μM (*SI Appendix*, Table S1 and Figs. S3 and S4) with no signs of cytotoxicity observed under the microscopy. A separate cell toxicity assay showed that bepridil was not toxic to Vero E6 and A549/ACE2 cells until its concentration reached above 25 and 50 μM, respectively (*SI Appendix*, Fig. S5). In order to characterize bepridil EC_50_ values in inhibiting SARS-CoV-2 in the two cell lines, both cells were treated with bepridil at different concentrations and infected with SARS-CoV-2 at a multiplicity of infection (MOI) of 0.5. Infected cells were then cultured for 3 d for Vero E6 and 4 d for A549/ACE2 cells before assessing the yields of infectious progeny virus. Based on the efficacy to inhibit SARS-CoV-2 infection, EC_50_ values were estimated to be 0.86 and 0.46 μM in Vero E6 and A549/ACE2 cells, respectively (Fig. 5). These values are similar to remdesivir’s reported EC_50_ value of 0.77 μM in Vero E6 cells (42). The strong inhibition of SARS-CoV-2−induced CPE in Vero E6 and A549 cells by bepridil at a concentration much lower than its IC_50_ value for inhibiting M^p^^ro^ is likely due to the aforementioned dual functions or other cellular effects of bepridil. In patients, bepridil can reach a state C_max_ as 3.72 μM (43). This concentration is effective in inhibiting SARS-CoV-2 based on our virus MN analysis. Collectively, our results indicate that bepridil is an effective medicine in preventing SARS-CoV-2 from entry and replication in mammalian cell hosts. Therefore, we urge the consideration of clinical tests of bepridil in the treatment of COVID-19.

Bepridil was voluntarily withdrawn from the US market in 2004 due to its side effects such as QT prolongation (44). It has also been implicated that its usage might cause ventricular arrhythmia (45). Despite those potential drawbacks, bepridil is still marketed in Japan, China, and France. Several factors, such as its in vivo efficacy against SARS-CoV-2 and cardiovascular effects of COVID-19, need to be assessed before attempting to explore clinical benefits of using it in COVID-19 patients.

## Conclusion

Guided by a computational docking analysis, we experimentally characterized about 30 FDA/EMA-approved drugs on their inhibition of the essential M^p^^ro^ enzyme of the COVID-19 pathogen SARS-CoV-2. From the study, we identified six FDA/EMA-approved drugs that can potently inhibit M^p^^ro^ with an IC_50_ value lower than 100 µM. One medicine, bepridil, exhibited strong inhibition of SARS-CoV-2 from entry and replication inside Vero E6 and A549 cells at a low micromolar concentration. Although the IC_50_ value for bepridil (72 µM) is relatively high, the better indicator of the potency of an antiviral drug candidate is its potency against live virus. Given that bepridil has been previously demonstrated to show efficacy in Ebola infected mice, we urge a serious consideration of its clinical tests in treating COVID-19. Our current study indicates that there is a large amount of FDA/EMA-approved drug space open for exploration that could hold promise for repurposing existing drugs to target COVID-19. Performing screening studies on different SARS-CoV-2 protein targets is necessary to uncover existing medicines that may be combined for mixture treatments of COVID-19. More explorations in this direction are imperative.

## Materials and Methods

### Chemicals.

We purchased econazole nitrate, duloxetine hydrochloride, doxapram hydrochloride monohydrate, clemastine fumarate salt, sertaconazole nitrate, isavuconazole, rupintrivir, and zopiclone from Sigma-Aldrich; pimavanserin, trihexyphenidyl hydrochloride, reboxetine mesylate, sertindole, bepridil hydrochloride, darunavir, nelfinavir mesylate, indinavir sulfate, lopinavir, tipranavir, saquinavir, pirenzepine hydrochloride, oxiconazole nitrate, pimozide, and rimonabant from Cayman Chemicals; ebastine and itraconazole from Alfa Aesar; metixene hydrochloride hydrate and lemborexant from MedChemExpress; fexofenadine hydrochloride from TCI Chemicals; ketoconazole from Acros Organics; clotiapine from Enzo Life Sciences; and oxyphencyclimine from Boc Sciences. We acquired Sub3 with the sequence as DABCYL−Lys−Thr−Ser−Ala−Val−Leu−Gln−Ser−Gly−Phe−Arg−Lys−Met−Glu−EDANS from Bachem Inc. DABCYL is the abbreviated form of 4-(dimethylaminoazo)benzene-4-carboxylic acid and EDANS is the abbreviated form of 5-((2-Aminoethyl)amino)naphthalene-1-sulfonic acid.

### Docking.

Autodock 4 was used for all docking analysis. For each small molecule, the genetic algorithm-based calculation was carried out for 100 runs, with each run having a maximal number of evaluations of 2,500,000.

### M^pro^ Expression and Purification.

We constructed the plasmid pBAD-sfGFP-M^pro^ from pBAD-sfGFP. The M^p^^ro^ gene was inserted between DNA sequences that coded sfGFP and 6xHis. The overall sfGFP-M^p^^ro^-6xHis fusion gene was under control of a pBAD promoter. The antibiotic selection marker was ampicillin. To express sfGFP-M^p^^ro^-6xHis, *E. coli* TOP10 cells were transformed with pBAD-sfGFP-M^p^^ro^. A single colony was picked and grew in 5 mL of lysogeny broth medium with 100 µg/mL ampicillin overnight. The next day, we inoculated this starting culture into 5 L of 2xYT medium with 100 µg/mL ampicillin in five separate flasks at 37 °C. When the OD (optical density) reached 0.6, we added l-arabinose (working concentration of 0.2%) to each flask to induce protein expression at 37 °C for 4 h. Then, the cells were pelleted at 4,000 rpm at 4 °C, washed with cold phosphate-buffered saline and stored at −80 °C until purification. To purify the expressed protein, we resuspended frozen cells in 125 mL of buffer containing Tris pH 7.5, 2.5 mM dithiothreitol (DTT), and 1.25 mg of lysozyme. We sonicated resuspended cells using a Branson 250W sonicator with 1 s on, 4 s off, and a total 5-min 60% power output in two rounds. After sonication, we spun down the cellular debris at 16,000 rpm for 30 min at 4 °C. We collected the supernatant and recorded the volume. The whole-cell lysate analysis showed that almost all of the fusion protein was hydrolyzed to two separate proteins, sfGFP and M^p^^ro^. We were able to obtain an insignificant amount of M^p^^ro^ when Ni-NTA (nickel-nitrilotriacetic acid) resins were used for purification. Therefore, we did ammonium sulfate precipitation using the whole-cell lysate. This was done by the addition of a saturated ammonium sulfate solution at 0 °C. We collected the fraction between 30% and 40% of ammonium sulfate. We dissolved the collected fraction in buffer A (20 mM Tris, 10 mM NaCl, and 1 mM DTT at pH 8.0) and dialyzed the obtained solution against the same buffer to remove ammonium sulfate. Then, we subjected this solution to anion exchange column chromatography using Q Sepharose resins. We eluted proteins from the Q Sepharose column by applying a gradient with increasing concentration of buffer B (20 mM Tris, 1 M NaCl, and 1 mM DTT at pH 8.0). We concentrated the eluted fractions that contained M^p^^ro^ and subjected the concentered solution to size exclusion chromatography using a HiPrep 16/60 Sephacryl S-100 HR column with a mobile phase containing 10 mM sodium phosphate, 10 mM NaCl, 0.5 mM (ethylenedinitrilo)tetraacetic acid (EDTA), and 1 mM DTT at pH 7.8. The final yield of the purified enzyme was 1 mg/L with respect to the original expression medium volume. We determined the concentration of the finally purified M^pro^ using the Pierce 660-nm protein assay and aliquoted 10 µM M^p^^ro^ in the size exclusion chromatography buffer for storage at −80 °C.

### The Synthesis of Sub1.

We loaded the first amino acid (0.5 mmol, 2 equiv.) manually on chlorotrityl chloride resin (0.52 mmol/g loading) on a 0.25-mmol scale by the addition of N, N-Diisopropylethylamine (DIPEA) (3 equiv.). After addition of the first amino acid, automated Fmoc-based solid phases synthesis was performed using a Liberty Blue automated peptide synthesizer. Deprotection of the Fmoc group was carried out with 20% piperidine in dimethylformamide (DMF). Coupling was done with a Fmoc-protected amino acid (0.75 mmol, 3.0 equiv.) and the coupling reagent (1-[Bis(dimethylamino)methylene]-1H-1,2,3-triazolo[4,5-b]pyridinium 3-oxide hexafluorophosphate (HATU) (0.9 mmol, 3.6 equiv.) and DIPEA in N-Methyl-2-Pyrrolidone (1 mmol, 4.0 equiv.). The final amino acid was capped by the addition of 25% acetic anhydride (vol/vol) in DMF and DIPEA (0.2 mmol, 2.0 equiv.). Coumarin coupling was performed in anhydrous tetrahydrofuran (THF) using T3P in EtOAc (50% wt/vol) (3.0 equiv.), DIEPA (3 equiv.) and 7-amino-4-methyl-coumarin (0.8 equiv.) and mixed for 16 h. The solvent was removed, and the peptide was dissolved in dichloromethane (DCM) and washed with H_2_O (4×) followed by HCl (2×) and brine (1×). The organic layer was dried with Na_2_SO_4_, filtered, and concentrated. Global deprotection was then carried out using triisopropylsilane (5%) and trifluoroacetic acid (30%) vol/vol in DCM and mixed for 2 h to 3 h to result in the crude substrate. The peptide was then purified by semipreparative high performance liquid chromatography (HPLC), and the fractions containing pure product were pooled, concentrated, and analyzed by liquid chromatography–mass spectrometry (LC–MS) for purity.

### The Synthesis of Sub2.

We performed automated Fmoc-based solid phase synthesis on a Liberty Blue automated peptide synthesizer. Synthesis was conducted on a 0.1-mmol scale with Fmoc Rink amide 4-Methylbenzhydrylamine (MBHA) resin (0.52 mmol/g loading) and 3 equiv. of protected amino acids. Deprotection of the Fmoc group was carried out with 20% piperidine/DMF. Coupling was done using the desired Fmoc-protected amino acid (0.2 mmol, 2.0 equiv.), coupling reagent Oxyma (0.4 mmol, 4.0 equiv.), and N,N′-Diisopropylcarbodiimide (DIC) (0.4 mmol, 4.0 equiv.). After the final amino acid had been coupled on, the resin was washed twice with DMF and DCM. Cleavage from the resin was performed using trifluoroacetic acid (95%), triisopropylsilane (2.5%), and water (2.5%) with agitation for 4 h. The peptide was drained into cold methyl tert-butyl ether, where it precipitated out. We centrifuged the precipitate, decanted mother liquor, dissolved the pellet in DMF, and then purified the peptide by LC-MS.

### Screening Assay.

The 5-mM stock solutions of medicines were prepared in DMSO. The final screening assay conditions contained 50 nM M^p^^ro^, 10 µM Sub3, and 1 mM medicine. We diluted enzyme stock and substrate stock solutions using a buffer containing 10 mM sodium phosphate, 10 mM NaCl, and 0.5 mM EDTA at pH 7.8 for reaching the desired final concentrations. We ran the assay in triplicate. First, we added 30 µL of a 167-nM M^p^^ro^ solution to each well in a 96-well plate and then provided 20 µL of 5-mM stock solutions of medicines in DMSO. After a brief shaking, we incubated the plate at 37 °C for 30 min. Then we added 50 µL of a 20-µM Sub3 solution to initiate the activity analysis. The EDANS fluorescence with excitation at 336 nm and emission at 455 nm from the cleaved substrate was detected. We determined the fluorescence increasing slopes at the initial 5 min and normalized them with respect to the control that had no inhibitor provided.

### Inhibition Analysis.

The final inhibition assay conditions contained 50 nM M^p^^ro^, 10 µM Sub3, and a varying concentration of an inhibitor. Similar to screening assay, we diluted enzyme stock and substrate stock solutions using a buffer containing 10 mM sodium phosphate, 10 mM NaCl, and 0.5 mM EDTA at pH 7.8 for reaching the desired final concentrations. We ran the assay in triplicate. For the inhibition analysis, we added 30 µL of a 167-nM M^p^^ro^ solution to each well in a 96-well plate and then provided 20 µL of inhibitor solutions with varying concentrations in DMSO. After a brief shaking, we incubated the plate at 37 °C for 30 min. Then we added 50 µL of a 20-µM Sub3 solution to initiate the activity analysis. We monitored the fluorescence signal and processed the initial slopes in the same way described in screening assay part. We used GraphPad 8.0 to analyze the data and used the [Inhibitor] vs. response – Variable slope (four parameters) fitting to determine the values of both IC_50_ and Hill coefficient.

### SARS-CoV-2 Inhibition by a Cell-Based Assay.

A slightly modified standard live virus-based MN assay was used as previously described (46–49) to rapidly evaluate the drug efficacy against SARS-CoV-2 infection in Vero E6 and A549 cell culture. Briefly, confluent Vero E6 or A549/ACE2 cells grown in 96-well microtiter plates were pretreated with serially twofold diluted individual drugs in duplicate over eight concentrations for 2 h before infection with ∼100 and ∼500, respectively, infectious SARS-CoV-2 particles in 100 µL of Eagle's minimal essential medium (EMEM) supplemented with 2% fetal bovine serum (FBS). Vero E6 and A549 cells treated with parallelly diluted DMSO with or without virus were included as positive and negative controls, respectively. After cultivation at 37 °C for 3 d (Vero E6) or 4 d (A549/ACE2), individual wells were observed under a microscope for the status of virus-induced formation of CPE and cytotoxicity. The efficacy of individual drugs was calculated and expressed as the lowest concentration capable of completely preventing virus-induced CPE in 100% of the wells with no signs of cytotoxic effect. All compounds were dissolved in 100% DMSO as 10-mM stock solutions and diluted in culture media. The toxicity to the treated cells was assessed by the Cell Cytotoxicity Assay kit (Abcam Cat#ab112118) according to the manufacturer’s protocol.

To quantify EC_50_ values, Vero E6 and A549/ACE2 cells grown in 24-well plates were pretreated with serially twofold diluted drug for 2 h before infection with SARS-CoV-2 at an MOI of 0.5 in 200 μL EMEM supplemented with 2% FBS. Cells treated with parallelly diluted DMSO with or without virus were included as positive and negative controls, respectively. After incubation for an hour with viral inoculum, cells were washed three times with EMEM, and cultivated with fresh medium for 3 d (Vero E6) or 4 d (A549/ACE2). Supernatants from infected cells were harvested for measuring the infectious virus titers by the tissue culture infective dose (TCID50) assay using Vero E6 cells. Briefly, 50 μL supernatants from infected cells were serially diluted (10-fold) in EMEM supplemented with 2% FBS; 100 μL of serially diluted samples were added to VeroE6 cells grown in 96-well plates and cultivated at 37 °C for 3 d followed by observation under a microscope for the status of virus-induced formation of CPE in individual wells. The titers were expressed as log TCID50/mL

## Supplementary Material

Supplementary File

## Data Availability

All study data are included in the article and/or *SI Appendix*.
