# Supplementary material for: Bepridil is potent against SARS-CoV-2 in vitro
Source: Proc Natl Acad Sci U S A. 2021 Feb 17;118(10):e2012201118. doi: 10.1073/pnas.2012201118 (PMC7958448; doi:10.1073/pnas.2012201118)
Supplement: Supplementary File [file pnas.2012201118.sapp.pdf]

Supplementary Information for
Bepridil is Potent against SARS-CoV-2 In Vitro

Erol C. Vatansever, Kai S. Yang, Aleksandra K. Drelich, Kaci C. Kratch, Chia-Chuan Cho, Kempaiah Rayavara Kempaiah, Jason C. Hsu, Drake M. Mellott, Shiqing Xu, Chien-Te K. Tseng*, and Wenshe Ray Liu*

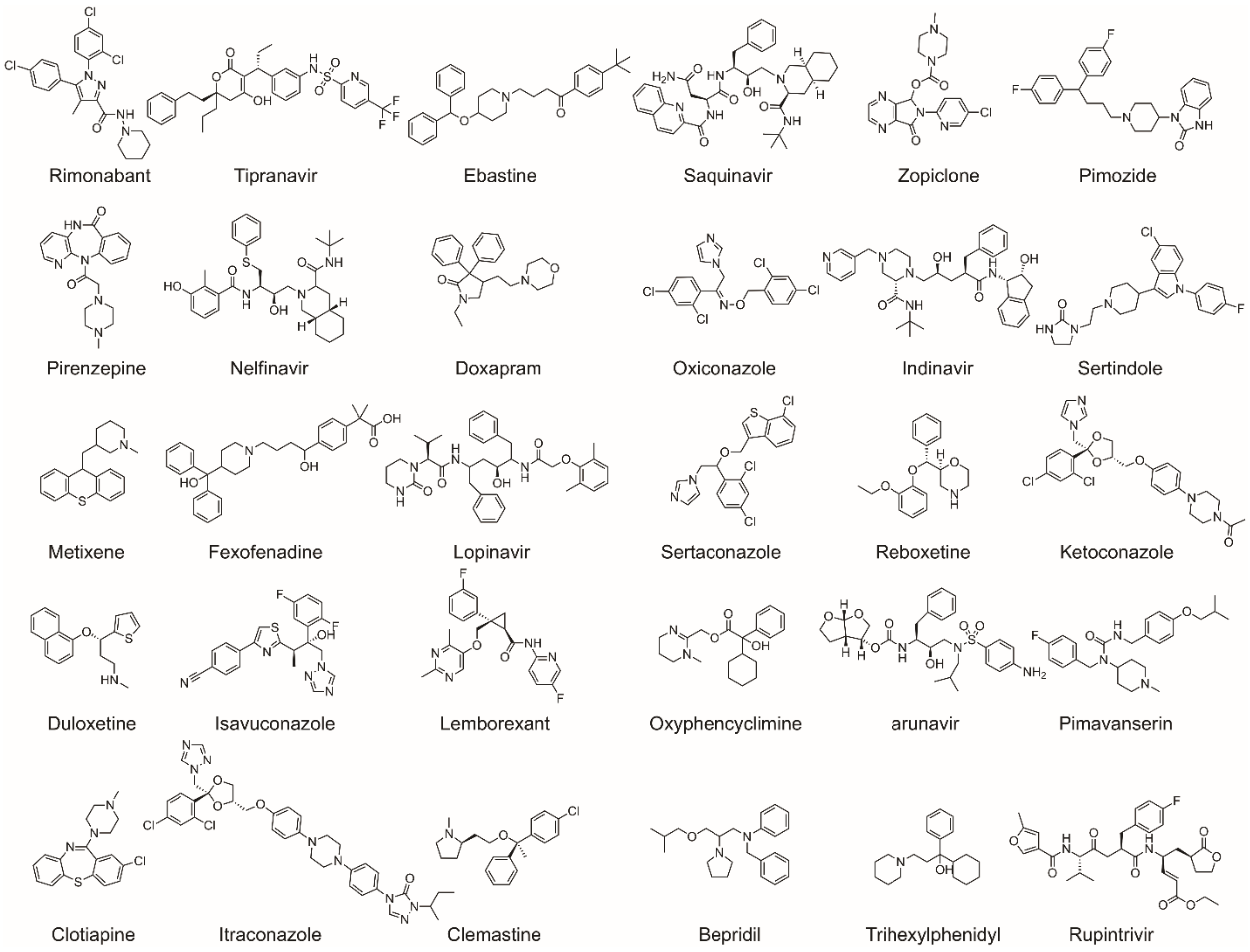

Fig. S1: Structures of 29 FDA/EMA-approved medicines and rupintrivir whose IC_{50} values in inhibiting M^{Pro} were determined in the study.

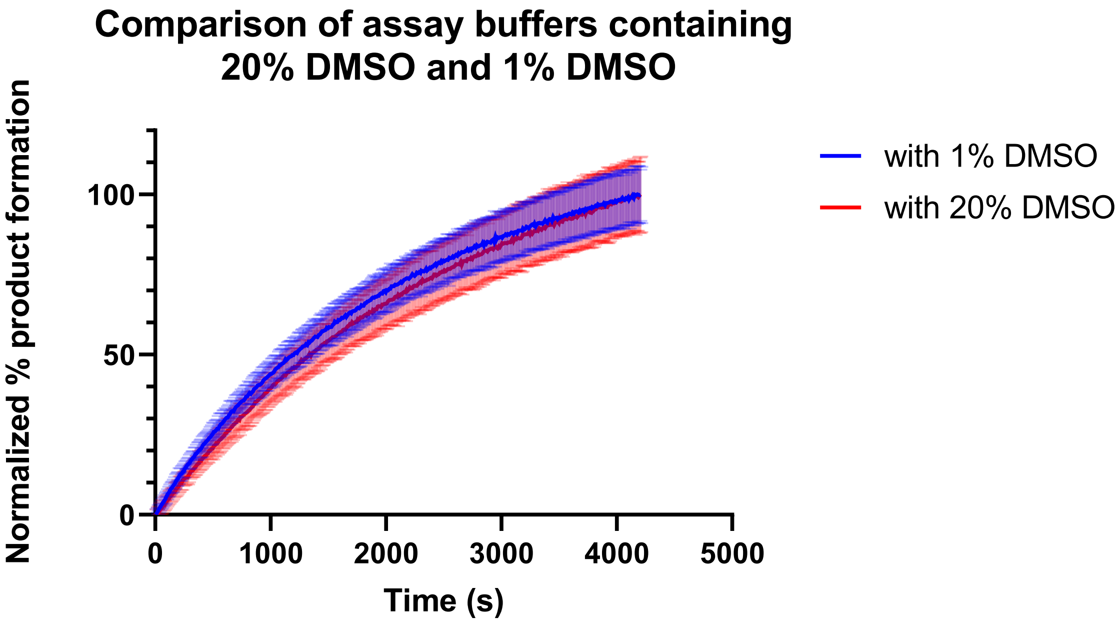

Fig. S2 Comparison of M^{Pro} activity in assay buffers containing 20% DMSO and 1% DMSO.

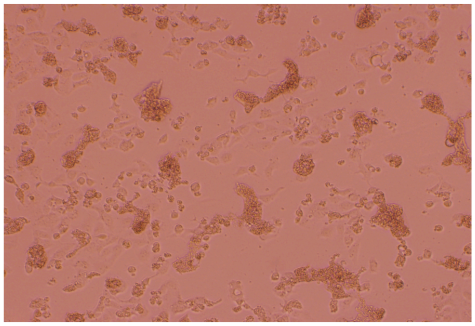

DMSO, Strong CPE

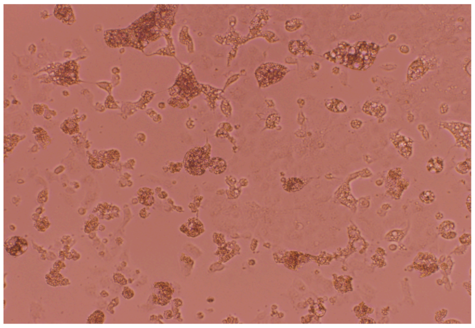

0.78 µM Bepridil, Strong CPE

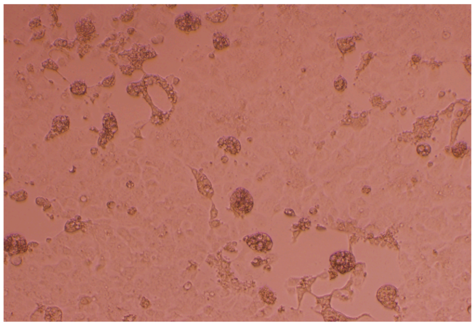

1.56 µM Bepridil, Strong CPE

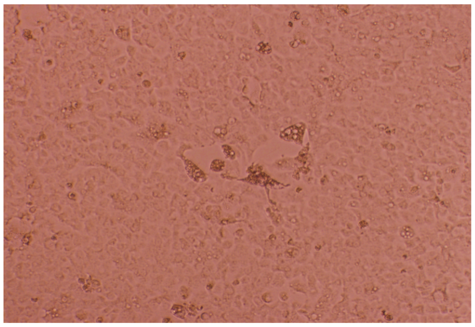

3.12 µM Bepridil, Single CPE

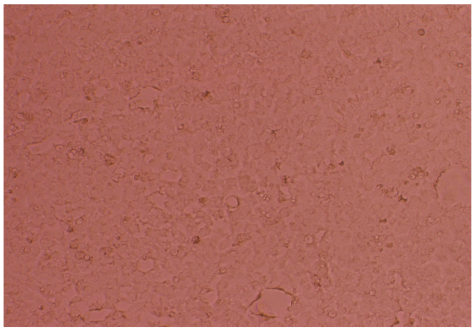

6.25 µM Bepridil, No CPE

Fig. S3: Microscope-recorded cytopathogenic effect (CPE) observation in Vero E6 cells that were infected by SARS-CoV-2 and grown in the presence of different concentration of bepridil or 0.1% DMSO as a positive control. Experimental conditions: Confluent Vero E6 cells grown in 96-wells microtiter plates were treated with various concentrations of bepridil before infection with ~100 infectious SARS-CoV-2 particles in 100 µL EMEM supplemented with 2% FBS. Cells treated with 0.1% DMSO and virus were included as positive control. After cultivation at 37 °C for 3 days, individual wells were observed under the microcopy for the status of virus-induced formation of CPE. Concentrations above 6.25 µM led to a same result as 6.25 µM and therefore are not shown.

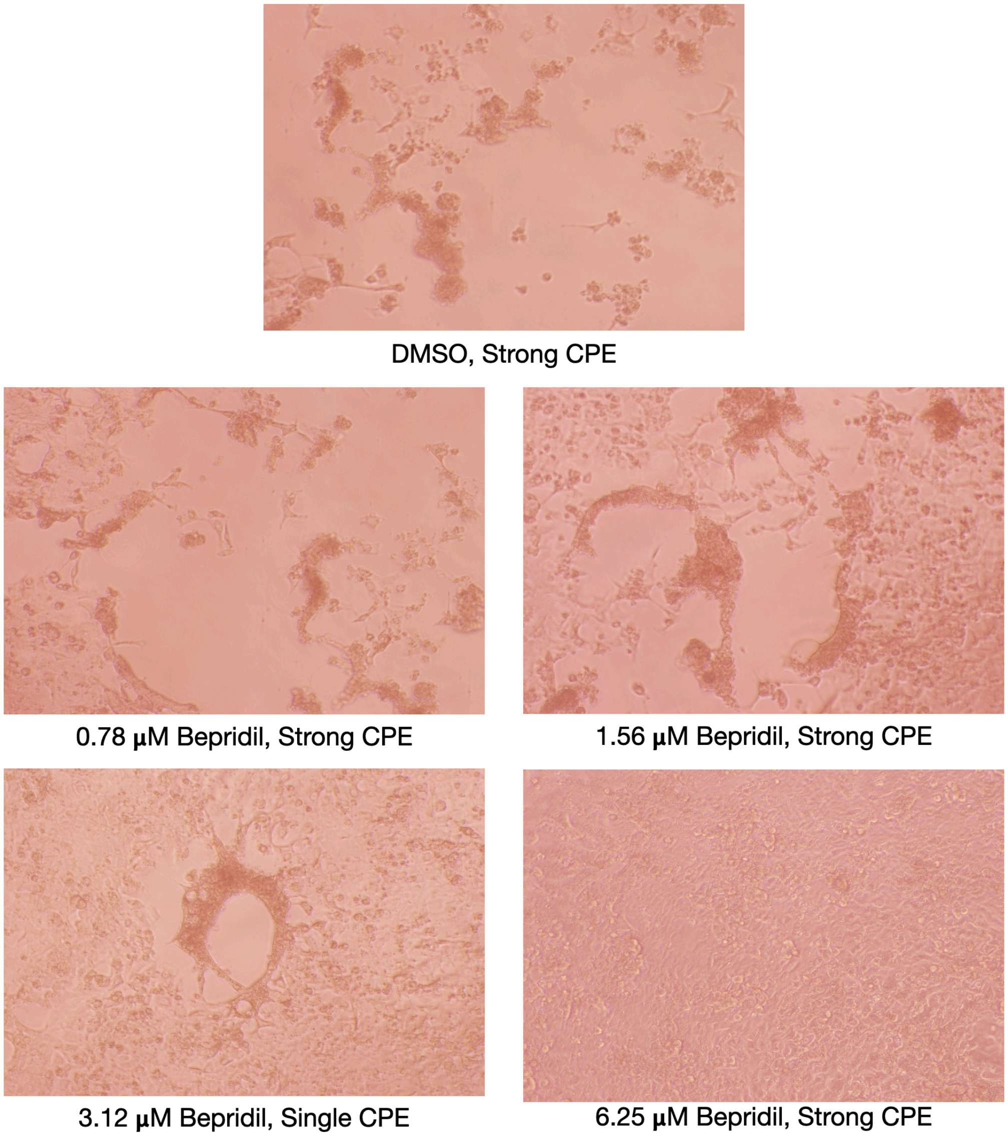

Fig. S4: Microscope-recorded cytopathogenic effect (CPE) observation in A549/ACE2 cells that were infected by SARS-CoV-2 and grown in the presence of different concentration of bepridil or 0.1% DMSO as a positive control. Experimental conditions: Confluent A549/ACE2 cells grown in 96-wells microtiter plates were treated with various concentrations of bepridil before infection with ~500 infectious SARS-CoV-2 particles in 100 µL EMEM supplemented with 2% FBS. Cells treated with 0.1% DMSO and virus were included as positive control. After cultivation at 37 °C for 4 days, individual wells were observed under the microcopy for the status of virus-induced formation of CPE. Concentrations above 6.25 µM led to a same result as 6.25 µM and therefore are not shown.

Table S1: SARS-CoV-2 induced CPE in (A) Vero E6 and (B) A549/ACE2 cells in the presence of bepridil

A. Vero E6 cells

Bepridil (μM)	Repeat #1	Repeat #2	Repeat #3	Repeat #4	Repeart #5	Repeat #6
25	No CPE	No CPE	No CPE	No CPE	No CPE	No CPE
12.5	No CPE	No CPE	No CPE	No CPE	No CPE	No CPE
6.25	No CPE	No CPE	No CPE	No CPE	No CPE	No CPE
3.125	CPE	CPE	CPE	No CPE	CPE	CPE
1.56	CPE	CPE	CPE	CPE	CPE	CPE
0.78	CPE	CPE	CPE	CPE	CPE	CPE

B. A549/ACE2 cells

Bepridil (μM)	Repeat #1	Repeat #2	Repeat #3	Repeat #4	Repeart #5	Repeat #6
50	No CPE	No CPE	No CPE	No CPE	No CPE	No CPE
25	No CPE	No CPE	No CPE	No CPE	No CPE	No CPE
12.5	No CPE	No CPE	No CPE	No CPE	No CPE	No CPE
6.25	No CPE	No CPE	No CPE	No CPE	No CPE	No CPE
3.125	CPE	CPE	CPE	CPE	CPE	CPE
1.56	CPE	CPE	CPE	CPE	CPE	CPE
0.78	CPE	CPE	CPE	CPE	CPE	CPE

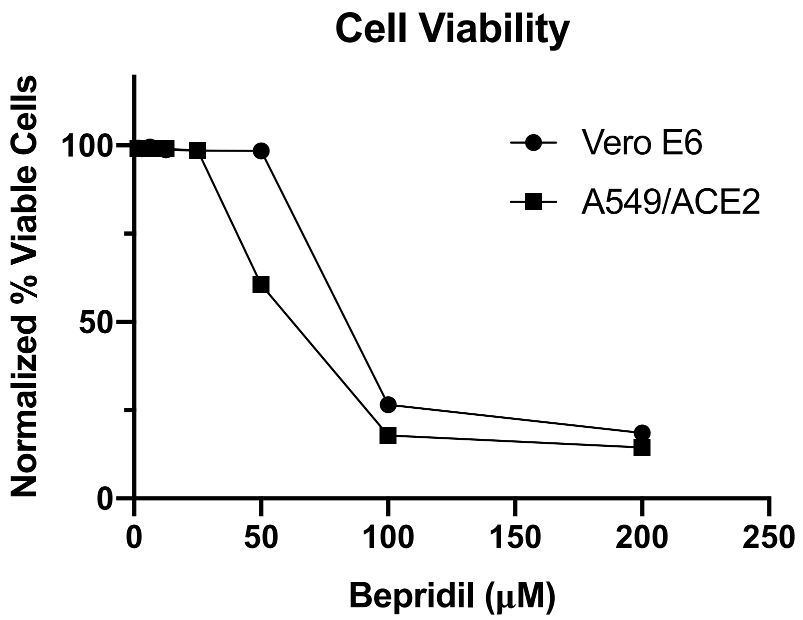

Fig. S5: Viability of Vero E6 and A549/ACE2 cells at different concentrations of bepridil.
